# General anesthesia with remimazolam for a pediatric patient with MELAS and recurrent epilepsy: a case report

**DOI:** 10.1186/s40981-022-00564-x

**Published:** 2022-09-16

**Authors:** Yusuke Yamadori, Yuki Yamagami, Yukihisa Matsumoto, Mari Koizumi, Akiyo Nakamura, Daiskuke Mizuta, Kyoko Yasuda, Gotaro Shirakami

**Affiliations:** 1 Department of Anesthesiology, Takamatsu Red Cross Hospital, 4-1-3 Bancho, Takamatsu City, Kagawa 760-0017 Japan; 2 Department of Anesthesia and Pain Medicine, Kagawa University Hospital, 1750-1 Ikenobe, Miki-cho, Kagawa 761-0793 Japan

**Keywords:** MELAS, Mitochondrial Encephalomyopathy, Remimazolam, General anesthesia, Epilepsy, Epileptic seizure, Open gastrostomy

## Abstract

**Background:**

Mitochondrial encephalomyopathy, lactic acidosis, and stroke-like episodes (MELAS) is a mitochondrial disease. We report here the safe use of remimazolam in a pediatric MELAS patient.

**Case presentation:**

A 10-year-old girl (118 cm, 16 kg) was scheduled for an open gastrostomy to improve nutrition and epileptic seizure control. We induced and maintained general anesthesia with remimazolam, remifentanil, fentanyl, and rocuronium. We also performed a bilateral subcostal transversus abdominis plane block before the surgery. The surgery finished uneventfully. After we discontinued remimazolam administration, the patient woke up immediately but calmly without flumazenil. Epileptic seizures did not occur during intra- and early post-operative periods.

**Conclusion:**

Remimazolam enabled us to provide a pediatric MELAS patient with general anesthesia without causing delayed emergence or epileptic seizures.

## Background

Mitochondrial encephalomyopathy, lactic acidosis, and stroke-like episodes (MELAS) is a rare mitochondrial disease (16–18 per 100,000) [1, 2]. MELAS presents various clinical manifestations: stroke-like episodes, epilepsy, dementia, recurrent headaches, hearing impairment, myopathy, lactic acidemia, diabetes, cardiac conduction abnormalities, cardiomyopathy, and short stature [1, 2]. Because of the infrequency of MELAS and the lack of prospective studies, anesthetic management of MELAS must rely on recommendations from case reports. Previous reports on mitochondrial disease have demonstrated several anesthetic hazards, including malignant hyperthermia associated with inhaled anesthetics [3, 4] and propofol infusion syndrome (PRIS) [5]. Furthermore, seizure-like activities induced by sevoflurane have been reported, especially in children [6]. The choice of anesthetic agents for patients with MELAS and epilepsy remains controversial.

Remimazolam is an ultrashort-acting benzodiazepine with similar pharmacological properties to midazolam, including anti-epileptic and less circulatory depression effects, and has a specific antagonist (flumazenil) [7–9]. To our knowledge, this is the first report on the use of remimazolam in a pediatric MELAS patient. This case report follows the CARE guidelines [10].

## Case presentation

A 10-year-old girl (height, 118 cm; weight, 16 kg) had been treated regularly by a doctor for autistic disorder since she was 2 years old. She had epilepsy since 6 years of age and was diagnosed as MELAS by genetic testing, which indicates the point mutation in mitochondrial DNA (3243A to G mutation). She had hyperlactatemia (preoperative blood lactate level 25 mg/dL; normal range 4–16 mg/dL), gastrointestinal symptoms (repeated constipation and vomiting), Wolff–Parkinson–White syndrome, and short stature. Due to progressive loss of motor skills and mental retardation, oral intake and medication adherence became difficult, including placement of peripheral venous catheters and feeding tubes. Therefore, feeding tubes were inserted once or twice daily at home or outpatient clinic to administer a small amount of nutrition and medication, including anti-epileptics (lacosamide and zonisamide). Her condition was unstable, and she experienced recurrent clonic seizures lasting 30 s to 1 min, several times a day. To improve her nutritional and epileptic situations, an open gastrostomy was scheduled.

General anesthesia (GA) was required because her cooperation could not be expected. Considering the risks reported [3–6], it was better to avoid inhaled anesthetics and propofol, if possible. Previous reports demonstrated that midazolam is safe as a general anesthetic in patients with mitochondrial diseases [11]. Midazolam has an anti-epileptic action; however, it can cause delayed awakening and respiratory depression because of its long half-life [12]. To avoid its disadvantages, we selected remimazolam, which has a significantly short half-life and potent anti-epileptic effect [7–9]. We planned to avoid the use of flumazenil because it could diminish the anti-epileptic action of benzodiazepines and complicate postoperative management. Premedication was not planned because of difficulties regarding its administration. Remifentanil and fentanyl were selected for intraoperative analgesia and rocuronium for muscle relaxation because they are considered safe for patients with mitochondrial diseases [11]. To reduce perioperative opioid use, a bilateral subcostal transversus abdominis plane (TAP) block was planned. We intended to measure the depth of anesthesia by bispectral index (BIS) monitor (186-0195-NK, Covidien, Minneapolis, MN, USA) with a target BIS value of 40 and that of muscle relaxation by electromyographic module (AF-101P, Nihon-Kohden, Tokyo, Japan) with a target value of train-of-four (TOF) count 1 or less. We selected saline as an intraoperative infusion fluid to prevent exacerbation of hyperlactatemia [13, 14] and precipitation that is formed by the combination of remimazolam and Ringer’s lactate/acetate solution [15]. The use of a warm-air heating device was designed to maintain body temperature during anesthesia and prevent shivering-induced hyperlactatemia and hypothermia-induced mitochondrial dysfunction [16].

A peripheral venous catheter and pulse oximeter were placed in the operating room. Following preoxygenation, GA was induced with remimazolam bolus 0.2 mg/kg, followed by a continuous infusion of 2.0 mg/kg/h and remifentanil 0.3 μg/kg/min. Monitoring of noninvasive blood pressure, electrocardiogram, BIS, and TOF (ulnar nerve stimulation) was initiated after loss of consciousness. Rocuronium at a dose of 0.3 mg/kg extinguished the TOF response, and trachea was intubated without coughing. Bilateral subcostal TAP block (0.375% ropivacaine 12 mL; 2.8 mg/kg) was performed after tracheal intubation. Remimazolam was reduced gradually from 2.0 to 1.0 mg/kg/h; however, BIS value remained approximately 30. Remifentanil dose was adjusted to surgical stimulation between 0.1 and 0.25 μg/kg/min. Fentanyl bolus at a dose of 25 μg (1.5 μg/kg) was administered intravenously during surgery. Additional rocuronium was administered to maintain TOF count 1 or 0. The procedure was completed uneventfully. The duration of the surgery was 73 min. Total doses of remimazolam, remifentanil, fentanyl, and rocuronium were 48.5, 0.38, 0.025, and 15.0 mg, respectively. Administration of sugammadex 2 mg/kg confirmed TOF ratio > 0.9. Nineteen minutes after the discontinuation of remimazolam and remifentanil administrations, spontaneous breathing and eye-opening were observed, and trachea was extubated. The patient was calm after extubation but hated wearing an oxygen mask. She was not shivering and was in stable oxygen saturation (approximately 97%) with room air breathing. She was subsequently transferred to the ward without oxygen mask under pulse oximeter monitoring. Nine hours after surgery (11 h after TAP block), she experienced pain at the gastrostomy site, but it was improved by intravenous administration of acetaminophen at a dose of 150 mg (9.4 mg/kg). Epileptic seizure did not occur during intraoperative and immediate post-operative periods. The control of epilepsy was improved by medications through the gastrostomy, which started the day after surgery; occasional epileptic seizures were noted after postoperative day 5, but the frequency was drastically reduced compared to that before surgery.

## Discussion

The selection of general anesthetics for patients with mitochondrial diseases is a serious concern due to anesthesia-associated risks, including malignant hyperthermia related to inhaled anesthetics or depolarizing muscle relaxants [3, 4], PRIS [5], hyperlactatemia [13, 14], and increased sensitivity to non-depolarizing muscle relaxants [17]. Most pediatric anesthesiologists in the USA choose inhaled anesthetics for GA because there is no clear evidence that they increase the risk of malignant hyperthermia in these patients [11]. Another report recommends propofol over inhaled anesthetics in patients with mitochondrial diseases because perioperative lactic acidosis occurs more frequently with inhaled anesthetics than with propofol [14]. Moreover, propofol is anti-epileptic, but some types of inhaled anesthetics are pro-epileptic [6]. Since propofol can depress mitochondrial function and cause PRIS [5], it should be administered carefully, especially in large doses or long-term administration. Compared to propofol, midazolam (benzodiazepine) has several advantages, including less circulatory depression, less injection pain, and has a specific antagonist (flumazenil) [7–9]. Benzodiazepines, as well as propofol, have an anti-epileptic effect. GA with midazolam is reportedly safe in patients with mitochondrial diseases [11]. However, because of its long half-life, it can cause delayed awakening and long-lasting respiratory depression [12]. Flumazenil can antagonize midazolam but also cause re-sedation and serious side effects, including epileptic seizures, by diminishing the benzodiazepine-related desirable effects [12].

Remimazolam may be a good choice in patients with mitochondrial diseases because it has midazolam-like pharmacological effects and an ultra-short-acting property that can avoid flumazenil use. Flumazenil can cause re-sedation after the antagonism of remimazolam [18–20] as well as midazolam [12]. In our report, the patient could wake up relatively soon (19 min) after remimazolam discontinuation without flumazenil use. Since she had low BIS values (approximately 30) intraoperatively, she might awaken more quickly if a lower dose of remimazolam were used and higher BIS values had been maintained. Although she could not tolerate wearing the oxygen mask after extubation, she revealed good oxygenation without oxygen supplement, indicating that remimazolam can awake fully soon and has no long-lasting respiratory depression effect.

Opioids and local anesthetics (regional anesthesia) are considered to be safe in patients with mitochondrial diseases [11]. In our case, intraoperative analgesia was provided by remifentanil, fentanyl, and TAP block. TAP block provided good post-operative analgesia presumably because the painful site was localized to the gastrostomy area. No requirement for post-operative opioids may be another reason for no postanesthesia respiratory depression.

Patients with mitochondrial diseases may have increased sensitivity to nondepolarizing muscle relaxants [17]. In this case, rocuronium at the dose of 0.3 mg/kg extinguished the ulnar nerve-stimulated twitch response and cough reflex during intubation. In ordinary adults, this dose is the 95% effective dose (ED95) in the adductor pollicis muscle but less than ED95 in the diaphragm (0.5 mg/kg) [21]. This fact might represent the patient’s increased sensitivity to rocuronium; however, there was no other apparent evidence of increased sensitivity, because the intraoperative rocuronium requirement was almost the same as usual.

In August 2022, we searched the PubMed database using the following keywords: <remimazolam> AND <MELAS> or <mitochondrial disease>. The results yielded two case report articles [22, 23], neither of which reported on pediatric cases. Since MELAS is a very rare disease, further reports are desirable for more appropriate anesthetic management.

In conclusion, we safely managed GA with remimazolam in a pediatric MELAS patient undergoing open gastrostomy. No intraoperative or early postoperative epileptic seizures occurred. Remimazolam could be a new anesthetic option for MELAS patients with epilepsy.

## Data Availability

Not applicable.

## Abbreviations

MELAS: Mitochondrial encephalomyopathy, lactic acidosis, and stroke-like episodes
PRIS: Propofol infusion syndrome
GA: General anesthesia
TAP: Transversus abdominis plane
BIS: Bispectral index
TOF: Train-of-four
ED95: 95% effective dose
